# The Effect of Light Intensity, Temperature, and Oxygen Pressure on the Photo-Oxidation Rate of Bare PbS Quantum Dots

**DOI:** 10.3390/nano8050341

**Published:** 2018-05-18

**Authors:** Huiyan Liu, Qian Dong, Rene Lopez

**Affiliations:** 1 School of Physical Science and Technology, ShanghaiTech University, 393 Middle Huaxia Road, Shanghai 201210, China; liuhy3@shanghaitech.edu.cn; 2 Shanghai Institute of Ceramics, Chinese Academy of Sciences, Shanghai 200050, China; 3 University of Chinese Academy of Sciences, Beijing 100049, China; 4 Department of Applied Physical Sciences, University of North Carolina at Chapel Hill, Chapel Hill, NC 27599, USA; qdong@live.unc.edu

**Keywords:** PbS, quantum dots, photo-oxidation, kinetics of oxidation, photoluminescence

## Abstract

The oxidation speed of PbS quantum dots has been a subject of controversy for some time. In this study, we reveal the precise functional form of the oxidation rate constant for bare quantum dots through analysis of their photoluminescence as a function of temperature, oxygen pressure, and excitation-laser intensity. The combined effect of these factors results in a reduced energy barrier that allows the oxidation to proceed at a high rate. Each absorbed photon is found to have a 10^−8^ probability of oxidizing a PbS atomic pair. This highlights the importance of photo-excitation on the speed of the oxidation process, even at low illumination conditions. The procedure used here may set up a quantitative standard useful for characterizing the stability of quantum dots coated with ligands/linkers, and to compare different protection schemes in a fair quantitative way.

## 1. Introduction

Lead sulfide (PbS) quantum dots (QDs) continue to be a material of large scientific and technological interest due their size-dependent optical properties. Above all, PbS QDs present a remarkably tunable optical absorption and luminescence from the visible to the near-infrared range. This particular optical property, coupled with its charge transport characteristics, have led to PbS QDs being considered and already advantageously exploited in many electro-photonic applications, including filed-effect transistors (FETs) [1,2,3], solar cells [4,5,6], and photo detectors [7]. QDs often present unsatisfied dangling bonds at the surface, which give rise to surface defect states in the bandgap. Despite the progress achieved by colloidal synthesis and the molecular surface passivation of those defects, one of the major obstacles that limits the scope of QD applications is the nature and kinetics of environmentally induced QDs chemical instabilities, because these usually lead to deleterious QD compositional changes and degradation of the material’s performance [8].

Several researchers have previously investigated the stability of PbS QDs [9,10]. Most of these inquiries have noted the existence of thermal [11,12] and photo-induced paths for oxidation and degradation [8]. However, in the vast majority of these studies, the physical and chemical changes to the chalcogenide surface are convoluted with the degradation or alterations in the capping or linking ligands, which mask the evolution of the PbS itself under the environmental variables of interest (light, gas and temperature). This is fair in some instances, as for many applications, those molecules are an integral part of the device operation. However, in no few cases, the environmental evolution of the QDs becomes intricately more complex, making it extremely hard to arrive at precise descriptions of the physical-chemical processes. For instance, Peterson et al. [13] found that the photo-oxidation rate of PbS quantum dots was size dependent; this is a very interesting result, but the QDs studied were oleic acid coated and embedded within a polymer, which made the gas-QD surface interaction hard to describe in a quantitative way. When Zhang et al. [14] observed photo-induced luminescence (PL) enhancement in similarly-prepared oleic acid coated PbS QDs in solution, they determined that the light activation was dependent on the solution concentration. However the QD’s surface change or its kinetics induced by the light could not be precisely quantified since the light intensity on the QDs varied with penetration depth and the solution was bound to present convection. Beyond lacking quantitative assessment, Turyanska et al. [15] studied the temperature dependence of the near-infrared PL emission from thiol-capped PbS QDs; finding that such PbS QDs showed no time-dependent change. In contrast, Ihly et al. [11] showed that ethanedithiol-treated PbS QDs in films will oxidize, ripen, and even sinter. Because most of these discrepancies could be traced to non-trivial differences in testing conditions and QDs cappings, to have a clear relationship between light, gas pressure, temperature, and the rate of change at the surface of PbS quantum dots, we propose it is necessary to test the evolution under those environmental variables in non-aggregated ligand-free PbS QDs, with the goal of arriving to precise quantitative reaction rates. The quantitative knowledge from this condition will help to establish a reliable basis upon which to compare all other modified QDs. In this work, we report on the quantitative relationship between the above-mentioned variables and the oxidation of bare PbS QDs directly fabricated by pulsed laser deposition. We found that under relatively intense illumination with photon energy larger than the bandgap, the kinetics of the oxidation process can be fitted to a minimal physical kinetic model to obtain a precise relationship between the environmental conditions and the oxidation rate constant of the process. In particular, we note that when subjected to illumination, the rate of the oxidation was boosted to a linear relationship with light intensity and O_2_ pressure. Furthermore, under illumination, the temperature of the environment was also played a significant role, with an apparent activation energy barrier of only 0.19 eV. These findings, obtained from easy-to-follow changes in the PL emission under controlled conditions, are significant because they highlight the key role that photo-excitation performs in the material transformation process and the substitutive roles that O_2_, light intensity, and temperature can play in the oxidation of PbS QDs.

## 2. Materials and Methods

**Figure 1:**
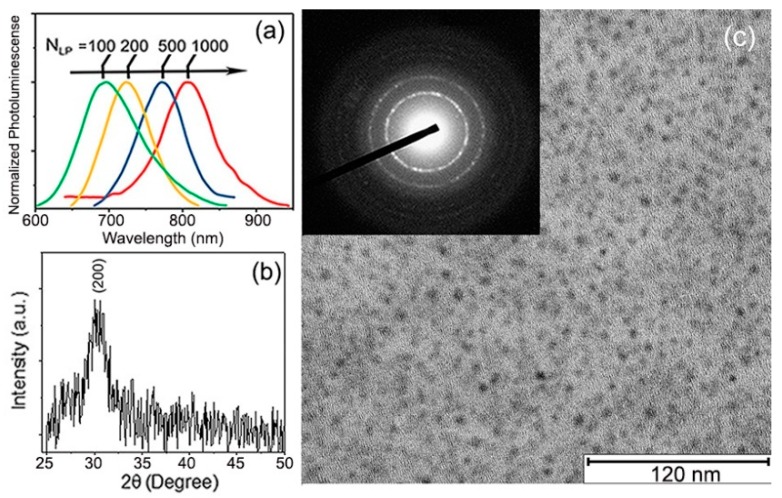
(**a**) Initial photoluminescence (PL) spectra of PLD-deposited PbS QDs with different number of laser shots (N_LP_). PL of the PbS QDs shows clearly red shift from 680 to 820 nm. This obvious shift is a consequence of the quantum size effects of the PbS QDs [16]; (**b**) XRD pattern of PbS QDs deposited with N_LP_ = 500; (**c**) TEM image of PbS QDs deposited directly onto carbon-filmed-grids; the inset in (**c**) is their TEM diffraction pattern.

Ligand-free PbS QDs were fabricated using pulsed laser deposition [16] (PLD) by ablation of a pressed PbS powder target using a KrF excimer laser (λ = 248 nm; repetition rate = 10 Hz, fluence = 5 J/cm^2^) under 500 mTorr of Helium (99.999% pure) background gas pressure that filled the chamber, which, prior to the experiment, was evacuated to $1\times 10^{-6}\;\mathrm{Torr}$. The PbS QDs growth was performed at room temperature onto fuse silica, SiO_2_/Si substrates, and transmission electron microscopy (TEM) carbon grids simultaneously and placed on a rotating holder parallel to, and 10 cm away, from the target. Although the method allowed for simple QDs size, controlled via the number of laser shots (N_LP_) (Figure 1a), we selected the samples made with N_LP_ = 500 shots to be the focus of this study to maintain a constant QD size. The QDs obtained under such conditions were ~2.5 nm in average diameter and ±1 nm enclosed the sample size dispersion. Figure 1b shows the X-ray diffraction pattern (XRD) obtained using a Bruker D8 Discover diffractometer (using Cu-Kα radiation) for samples deposited on the fuse silica substrates. With less than one monolayer of QDs, only one X-ray peak is easily distinguishable over the background. The position of this peak perfectly matched the (002) planes of cubic of PbS [17] (PDF card No. 05-0592). Figure 1c shows the TEM micrograph of these QDs, obtained using a JEOL 2100-plus microscope operated at 200 kV. The inset in Figure 1c displays the electron diffraction pattern for this sample, highlighting the crystallinity and random orientation of the QDs. The first few diffraction rings that could be distinguished with clarity corresponded to the (200), (220), and (222) crystal planes of PbS [18].

For this study, the primary tool used to track the evolution of the QDs was their PL. PL measurements were carried out by exciting the sample using 532 nm wavelength emissions from a continuous wave laser. Photoluminescence spectra were recorded by a thermoelectrically-cooled CCD camera. The system is integrated in a confocal microscope using a 50× microscope objective. However, important for our study, the laser instead of having a diffraction limited size was defocused so that the beam spot was at the sample and reached approximately 1.75 µm in radius. Thus, the area from which PL was detected by the collection photonic fiber (3 micrometer cores) was only the ~5% central top of the gaussian beam. This was done to secure a uniform intensity excitation over all the QDs under observation simultaneously. PL from QDs in the skirts of the gaussian beam excitation were not able to enter the microscope collection fiber.

## 3. Results

**Figure 2:**
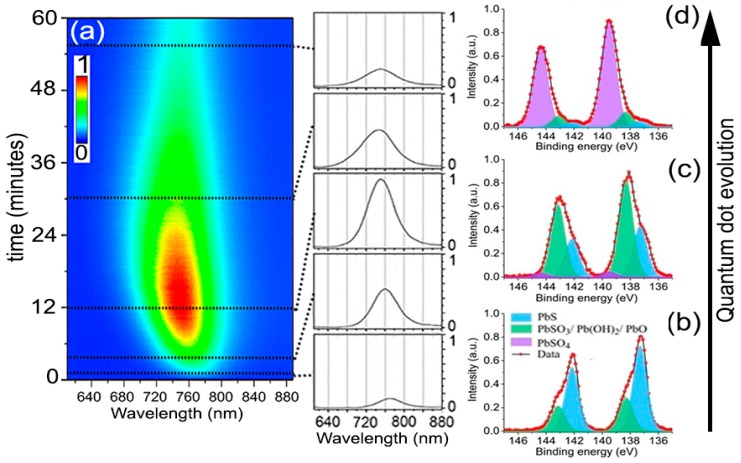
(**a**) Typical of the photoluminescence spectrum for the PbS QDs, all the experiments presented similar behavior but with marked time differences depending on the exact environmental conditions. (**b**–**d**) XPS Pb 4f_7/2_ spectrum of the freshly deposited, after laser-irradiation until reached maximum PL intensity, and after PL intensity decayed, respectively. The XPS measurement was conducted using a ThermoFisher ESCALAB 250XI Analyzer with base pressure below 10^−10^ mbar. Al Kα (1486.6 eV) radiation was used as an X-ray source (15 kV, 159.3 W).

Figure 2 shows the typical PL time evolution for all the experiments performed to analyze the oxidation kinetics of the QDs. The PL always started relatively weak with an emission peak centered at 765 nm wavelength. With time, the PL intensity increased progressively to eventually reach a maximum, for which the magnitude and time stability depended on the specific environmental variables and excitation intensity. Simultaneously, the PL peak could be observed shifting toward shorter wavelengths. After some additional time, lasting from a few seconds to several hours, still under continuous laser excitation, the intensity of the PL signal started to decrease toward low emission yields. Samples on SiO_2_ and SiO_2_/Si substrates behaved identically, ruling out possible significant heating effects from laser during this evolution. This process closely resembles the phenomenon of photoactivation extensively documented in CdSe nanocrystals [19,20,21]. Assuming our observations are fully analogous, the oxidation thus should follow a progressive reaction from the surface to the core, giving an initial PL enhancement through early surface oxide passivation, which continues deepening until the core of the QD is transformed. The chemical products of prolonged atmospheric oxidation of PbS QDs, albeit coated with molecular linkers, have been examined by several researchers [10,22,23]. Examination of the laser-irradiated spots (from beginning to end of their PL evolution) by fitting of the Pb 4f_7/2_ level obtained from X-ray photoelectron spectroscopy (XPS) shows that the chemical state of the Pb atoms can be deconvoluted into three major contributions (Figure 2b–d) of which relative weights vary with time. For the QDs freshly deposited and exposed to air but without any laser-irradiation, the XPS spectrum showed that, besides the 137 eV feature due to PbS, there was already a small but not negligible signal at 138.3 eV due to the initial formation of possible intermediate oxidation products [22,24], Pb(OH)_2_, PbO, and/or PbSO_3_. These products cannot be independently quantified here, although other researchers advocate that PbSO_3_ makes up the majority of the intermediates [10]. Inspection of areas where the experiment was stopped at the time of maximum PL intensity showed that the transformation of the PbS into the intermediate oxidation products had taken a significant fraction of the QDs’ mass (~60%), presumably just the most external atoms, reducing the QDs effective size to ~1.8 nm. Albeit a small fraction, this stage also seemed to correspond with the appearance of PbSO_4_, which marks the beginning of the PL decline. After a longer period under light exposure, when the PL decreased to a small fraction from its peak value, the Pb 4f_7/2_ peak shifted almost completely to 140.0 eV, indicating the bulk of each nanocrystal had been transformed into PbSO_4_ [24].

**Figure 3:**
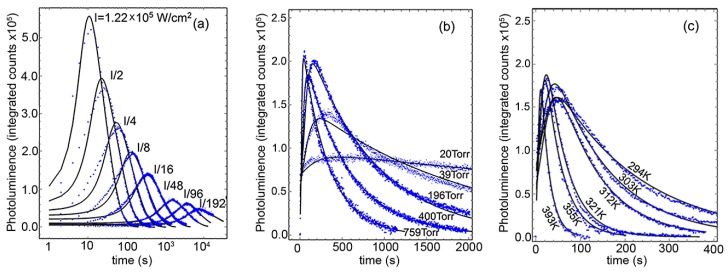
Time evolution of the photoluminescence integrated intensity for the PbS QDs, under three distinct experimental conditions: (**a**) Variation over laser power with 1 atmosphere of oxygen pressure and constant temperature (294 K), (**b**) variation under different oxygen pressures at room temperature and 1.54 × 10^4^ W/cm^2^ light intensity, and (**c**) temperature effect at constant pressure (1 atmosphere) and same constant laser power. Solid lines are fit to the physical model described in the text.

Figure 3 shows the all-wavelength-integrated PL as a function of time under the influence of the three environmental variables under consideration: Light intensity, O_2_ pressure, and temperature. As observed, the three variables had a direct effect on the rise and subsequent decrease of the total integrated PL counts, but its characteristic times and maxima require a detailed analysis to quantitatively obtain the overall kinetic properties of the oxidation process. To determine the specific mathematical form of the oxidation rate, we employ a simple phenomenological model of the PL under the oxidation process [25]. Here we suppose that in each QD and at all times, there exist *N_T_* constant pairs of PbS atoms that, under the oxidation process and illumination, can be found oxidized to some extent (*N_C_*), prepared to absorb a photon (*N_A_*), or already responsible for one exciton (N_A*_), such that *N_T_* = N_A_ + N_A*_ + N_C_. We do not specify each PbS pair location inside of the QD as it is assumed that the oxidation proceeds from the outside toward the center, that is, the *Nc* pairs make a growing outer shell at the expense of the decreasing N_A_ + N_A*_. Over time, the rate of change of each quantity should follow the following rate equations:

(1) $$\frac{dN_{A^{*}}}{dt}=\alpha \left(N_{A}-N_{A^{*}}\right)-\frac{N_{A^{*}}}{\tau }-k^{*}N_{A^{*}},$$

(2) $$\frac{dN_{A}}{dt}=\alpha \left(N_{A^{*}}-N_{A}\right)+\frac{N_{A^{*}}}{\tau }-kN_{A},$$

(3) $$\frac{dN_{C}}{dt}=k^{*}N_{A^{*}}+kN_{A},$$

where in α = σ*I/h*ν, *σ* represents the light absorption cross-sectional area per PbS pair, *I* is the intensity of the incident light, and *h*ν is the photon energy. *k* and k^*^ are the rate constants for the reaction under dark and light, respectively; and $\tau =\;{\left(\tau_{R}{}^{-1}+\tau_{NR}{}^{-1}\right)}^{-1}$ is the lifetime of the exciton allowing for non-radiative (*NR*) and radiative (*R*) paths to ground state decay. The first term in Equations (1) and (2) represents the absorption and stimulated emission, and the second term corresponds to the spontaneous emission. The third term in both Equations (1) and (2) represents the oxidation processes that result in an increase of the number of the oxidized pairs (*N_C_*), as is correspondingly shown in Equation (3).

**Figure 4:**
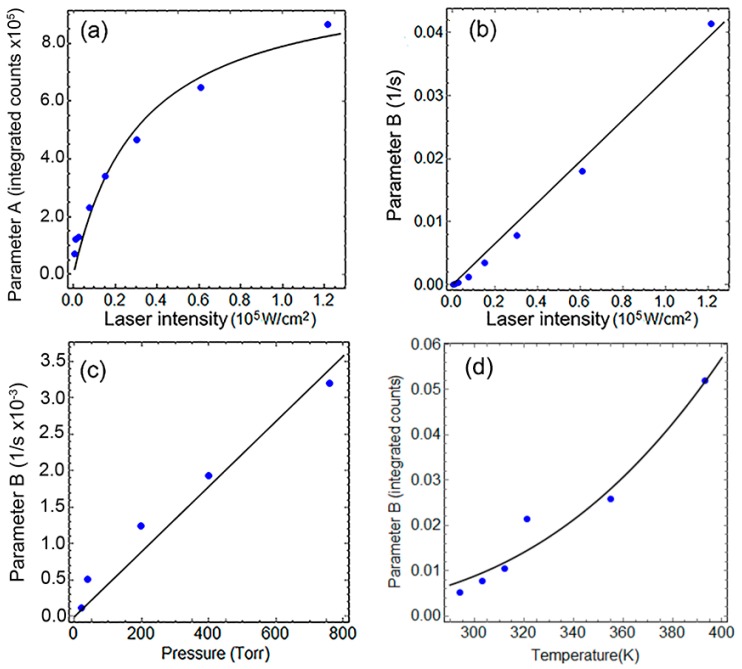
(**a**,**b**) parameters A and B under different light intensities at 294 K and 1 atmosphere of oxygen, respectively; (**c**) Parameter B as function of pressure at 294 K and 1.54 × 10^4^ W/cm^2^ light intensity; (**d**) Parameter B as function of temperature at 1 atmosphere and same light intensity. Dots are the specific parameters that produce the fitted curves in Figure 3. Solid lines are best fits to funcional forms of those parameters vs. the controlled enviromental variables as explained in detail in the Discussion section.

As the early oxidation of the surface has a PL-enhancing effect, we can expect that the initial oxidation lengthens the $\tau_{NR}$ to favor the radiative path of decay. Phenomenologically, we propose the non-radiative life time be inversely related to the number of non-oxidized PbS pairs as $\tau_{NR}\sim \frac{1}{{\left(N_{A^{*}}+N_{A}\right)}^{\gamma }}$, where γ is an exponent to be determined from the experiment. Because the radiative PL lifetime of PbS QDs has been measured by others and found to be ~1 µs [26], the reaction under light we measured in our experiments happens over ~1–1000 s, and the reaction under dark requires > 1 month [11], to explicitly solve the system of equations we adopted the following approximation $\tau^{-1}\gg \;k^{*}\gg k\;$~ 0. In this regime, the photon-mediated process is the only path for oxidation and the huge difference between $\tau^{-1}\gg \;k^{*}$ permit us to solve Equations (1) and (2), simultaneously, in a closed form to obtain quasi-static values for *N_A_* and N_A*_. The values of *N_A_* and N_A*_ will then slowly decrease under the rate defined in Equation (3) (detailed solution steps can be found in the Supplementary Information). Since PL ~ $\frac{N_{A}^{*}}{1+\tau_{R}/\tau_{NR}}$, this results in a rather simple explicit expression as the PL integrated counts as a function of time:

(4) $$PL\left(t\right)=A\frac{\exp\left(-Bt\right)}{\left(1+C\exp\left(-\gamma Bt\right)\right)},$$

with $A=M\left(\frac{\alpha }{\tau^{-1}+2\alpha }\right)$, $C=\frac{\tau_{R}}{\tau_{NR}{}_{t=0}}$, and the effective reaction rate $B=\frac{k^{*}\alpha }{\tau^{-1}+2\alpha }$. The model assumes all QDs are behaving similarly and does not include explicitly diffusion or any atomic-detail steps that must occur during the oxidation. As detailed work on metal nanoparticle oxidation has demonstrated [27], the complexity of a more detailed theory involves so large a number of parameters, such as a variety of activation energies, preexponential factors of individual oxidation stages, and ion and electron diffusion coefficients, that from the standpoint of the experimental observation, only their collective behavior could be probed. The simple model we employ (Equation (4)) contains just five parameters for determination, namely $M,\;\gamma ,C,\;\tau ,\;\mathrm{and}\;k^{*}$, with the rest derived from their relationships or fixed from the materials properties or experimental conditions (see the Supplementary Information). Of those five parameters, *M* is practically arbitrary and has no real importance, as it stands for the number of QDs in the examined area and the efficiency of the whole microscope optical train and detector setup. The remaining four are all physically meaningful, and although they all could in principle depend on the environmental variables, we can expect their sensitivity to them be quite low. τ, strictly speaking, should be a function of the oxidation, varying as the non-radiative recombination path is changing relevance. By this physical connection, all the environmental variables could affect τ, but in the approximation taken to solve for the quasi-static value of N_A*_, it is left to be a constant value, thus it represents an effective exciton lifetime, moving from non-radiative to radiative, over the whole QD oxidation process. The solid line in Figure 4a shows the fit of the A parameter from Equation (4), the amplitude coefficient of the PL counts. Following its functional form $A=M\left(\frac{\alpha }{\tau^{-1}+2\alpha }\right)$, we expect it to be strongly dependent on the light intensity alone. This allows the simultaneous determination of $M=2.08\times 10^{6}$ counts, and $\tau =6.11\times 10^{-8}\;s$. Although the model is fairly simple, τ results in the expected range between the best radiative life times reported and non-radiative values that would prevent PL emission.

Parameters γ and C work together to determine the onset behavior of the PL intensity increase. For all the samples and conditions explored, their fitting showed weak dependencies on the environmental variables (verified in the Supplementary Information). This perhaps means that all initial surface atom changes are all similarly fast, leaving the effective rate constant *B* to carry the weight of the environmental variables’ functionality. Figure 4c,d presents the functional fits of parameter *B* against laser power, O_2_ pressure, and temperature. Their functional form was not assumed before the fitting procedure, but as it can be seen, *B* presents a good simple linear dependence with the light intensity and the oxygen pressure, and an expected Arrhenius behavior with the absolute temperature. The collective information from those fits can be summarized in the following succinct expression, $B=B_{o}IP_{O_{2}}\exp\left(\frac{-\Delta E}{k_{B}T}\right)$, where I is the laser intensity in W/cm^2^, $P_{O_{2}}$ is the oxygen pressure in atmospheres (during the PL experiment, sample chamber was evacuated to 10^−5^ torr prior to filling with oxygen 99.999% pure), ΔE = 0.19 eV is the apparent energy barrier, $k_{B}$ is the Boltzman constant, *T* is the temperature, and $B_{o}=5.0\times 10^{-4}\frac{{\mathrm{cm}}^{2}}{s\cdot W\cdot atm}$ is the proportionality constant. To put in context how much lower the oxidation barrier appears under illumination, the free energy of reaction for formation of PbSO_4_ from PbS and O_2_ is reported to be −718 kJ/mol (−7.45 eV/PbS pair) [10]. Thus, illumination allows the oxidation to proceed at much higher rate.

## 4. Discussion

The effect of the light intensity on *B* has an interesting consequence, under 1 sun intensity ~0.1 W/cm^2^ for example, the process results in an effective rate of only $B=3.5\times 10^{-8}s^{-1}$, a significantly low value that, after early surface passivation, might give the appearance of very constant PL. This is in line with our experience and that from other researchers [11] whose QDs perform well even months after fabrication when stored in the laboratory air and exposed to ambient light. However, note this does not translate directly into the same behavior for $k^{*},$ the actual rate constant, which the model requires to be $k^{*}=2B_{o}P_{O_{2}}\exp\left(\frac{-\Delta E}{k_{B}T}\right)\;(I+h\nu /2\tau \sigma )$ (expression obtained from combining the above phenomenological expression for *B* and the model-required relationship with $k^{*}).$ This expression approaches a non-zero constant at low light intensities, which at 1 atmosphere and room temperature would be $k^{*}=2.07\times 10^{-2}\;s^{-1},$ a significantly faster rate than *B*. At low light intensities, the difference is the existence of an ατ factor in $B\approx \alpha \tau k^{*}$, the number of absorbed photons during one relaxation time. The implication of this difference is that the effective rate decreases as light intensity goes down, mostly because the number of attempts made by photons to oxidize the QD is low, but the reaction rate of those few absorbed photons remains relatively high. If, at best, the radiative life time is ~1 µs, then the oxidation reaction probability of an absorbed photon will be $\frac{k^{*}}{k^{*}+\tau^{-1}}$ ~$\;10^{-8}$. Therefore $k^{*}$ coupled with a large photon flux can bring oxidation very rapidly.

It is also worth remarking that low oxygen content effectively arrests the oxidation without need to reach extremely low pressures. At 20 mTorr we noticed the PL was activated and remained close-to-stable for many hours without significant change. However, crucially, at higher vacuum levels <10 mTorr, the PL yield did not increase at all, marking the environment quality required for a maximum oxygen concentration to protect QDs from oxidation under light utilization. Regarding the influence of temperature on the oxidation process, we can see that moderate above-room-temperature conditions readily enhanced the oxidation process. Due to some limitations in our microscope setup, we did not test low temperature evolution; however, given the functional form that fits our temperature data in the near-above-room-temperature range, only very low temperatures are likely to significantly slow down the oxidation process. On the contrary, under no-illumination conditions and full 1 atmosphere O_2_ pressure, the same temperature range probed in our experiment had not affected the QDs in any observable way, because after cooling the samples that experienced the thermal treatment under dark, showed PL behavior identical to samples that did not experience this treatment. Another observation we wish to mention is that performing the oxygen concentration experiments with “wet” oxygen (passing the gas through water before filling the chamber) did not change the kinetics at all, which does not support the conjecture that the presence of some water might aid the oxidation process significantly, in contrast to literature examples of QDs in solution where water is attributed an important role in the photo-oxidation [8].

## 5. Conclusions

The true rate constant for the oxidation of bare PbS QDs was determined through the fit of the PL integrated counts as function of temperature, oxygen pressure, and laser intensity, which highlighted their combined effect on the number photo-excited excitons and their reduced barrier to allow for the oxidation process. Although the oxidation steps and intermediate compounds are complicated, our experiment and model results seem to indicate that the overall oxidation reaction is first order with the oxygen pressure and the light intensity. Thus, even though the k^*^ is very large, the effective oxidation rate is dominated by the oxygen and photon availability. This work may help in setting a quantitative reference upon which characterize the stability of QDs in general, and specifically to encourage the study of the stability of those coated with ligands/linkers in a quantitatively precise and thus comparatively fair way. Identifying whether the source of protection against oxidation might come from blocking the photo-oxidation path, or by simply limiting the oxygen availability, might be important distinction in the design of increasingly more robust PbS QDs stabilization layers. With this distinction in mind, the precise effect of the relevant environmental variables on the changes of more complex QDs systems coated with other participating molecules could be assessed.

## Supplementary Materials

The following are available online at http://www.mdpi.com/2079-4991/8/5/341/s1.
