# Supplementary material for: The Effect of Light Intensity, Temperature, and Oxygen Pressure on the Photo-Oxidation Rate of Bare PbS Quantum Dots
Source: Nanomaterials (Basel). 2018 May 18;8(5):341. doi: 10.3390/nano8050341 (PMC5977355; doi:10.3390/nano8050341)

Supporting information

The effect of light intensity, temperature, and oxygen pressure on the photo-oxidation rate of bare PbS quantum dots

Huiyan Liu [1,2,3], Qian Dong [4] and Rene Lopez [1,4,*]

[1] School of Physical Science and Technology, ShanghaiTech University, 393 Middle Huaxia Road , Shanghai 20210, China
[2] Shanghai Institute of Ceramics, Chinese Academy of Sciences, Shanghai 200050, China
[3] University of Chinese Academy of Sciences, Beijing 101407, China
[4] Department of Applied Physical Sciences, University of North Carolina at Chapel Hill, Chapel Hill, North, Carolina 27599, United States
* Correspondence: rln@physics.unc.edu;

Section a) The Model for the oxidation is setup following the following 4 equations:

$$\begin{cases} N_A + N_A^* + N_C = N_T \\ \frac{dN_A^*}{dt} = \frac{\sigma I}{h\nu}(N_A - N_A^*) - \frac{N_A^*}{\tau} - k^* N_A^* \\ \frac{dN_A}{dt} = \frac{\sigma I}{h\nu}(N_A^* - N_A) - \frac{N_A^*}{\tau} - k^* N_A \\ \frac{dN_C}{dt} = k^* N_A^* + k N_A \end{cases}$$

Where N_A number of PbS atomic pairs,

N_A^* number of PbS exited pairs (or exciton number)

N_C number of PbS pairs transformed into a new oxide product that cannot produce photoluminescence (PL)

Additionally, the decay lifetime of the exciton

$$\tau^{-1} = \tau_R^{-1} + \tau_{NR}^{-1}$$

τ_R radiative lifetime of PbS exciton

τ_{NR} non-radiative lifetime of PbS exciton

Thus, the PL yield depends on the number of created excitons and the radiative and non-radiative lifetimes as:

$$PL \sim \frac{N_A^* * \tau_R^{-1}}{\tau_R^{-1} + \tau_{NR}^{-1}} = \frac{N_A^*}{1 + \tau_R/\tau_{NR}}$$

As the non-radiative lifetime depends inversely with the number of defects, we propose a power function with the number of non-oxidized atoms as:

$$\tau_{NR} \sim \frac{1}{\text{Defects}} \sim \frac{1}{(N_A + N_A^*)^{\gamma}} = \frac{1}{(N_T - N_C)^{\gamma}}$$

As the oxidation is slow compared to the time the QD requires to reach equilibrium between N_A and N_{A*}, to a first approximation the equation system is first solved taken $k^* << 1/\tau$. k is much smaller than k^*, thus we will use $k = 0$ from here on. Thus, we proceed to solve explicitly for the fast process of photoexcitation (with neglected oxidation that is expected to happen much slower). Note τ is approximated to be constant on this part of the solution.

$$\frac{dN_A^*}{dt} = \frac{\sigma I}{h\nu} N_A - \left(\frac{\sigma I}{h\nu} + \frac{1}{\tau}\right) N_A^*$$

$$\frac{dN_A}{dt} = -\alpha N_A + \beta N_A^*$$

$$\left(\alpha = \frac{\sigma I}{h\nu}\ ;\ \ \beta = \frac{\sigma I}{h\nu} + \frac{1}{\tau}\right)$$

→

$$\frac{d}{dt}\begin{pmatrix} N_A^* \\ N_A \end{pmatrix} = \begin{pmatrix} -\beta & \alpha \\ \beta & -\alpha \end{pmatrix} \begin{pmatrix} N_A^* \\ N_A \end{pmatrix}$$

$$\begin{pmatrix} N_A^* \\ N_A \end{pmatrix}_{t=0} = \begin{pmatrix} 0 \\ N_T - N_C \end{pmatrix}$$

↓

$$\det\begin{pmatrix} -\beta - \lambda & \alpha \\ \beta & -\alpha - \lambda \end{pmatrix} = (-\beta - \lambda)(-\alpha - \lambda) - \alpha\beta = 0$$

$$(\beta + \lambda)(\alpha + \lambda) - \alpha\beta = 0$$

$$\Rightarrow \alpha\beta + (\alpha + \beta)\lambda + \lambda^2 - \alpha\beta = 0$$

$$\lambda(\lambda + (\alpha + \beta)) = 0$$

characteristic values $\begin{cases} \lambda_1 = 0 \\ \lambda_2 = -(\alpha+\beta) \end{cases}$

For each eigenvalue, we obtain a characteristic vector

$\lambda = 0$

$$\begin{pmatrix} -\beta & \alpha \\ \beta & -\alpha \end{pmatrix}\begin{pmatrix} N_A^* \\ N_A \end{pmatrix} = \begin{pmatrix} 0 \\ 0 \end{pmatrix} \longrightarrow V_1 = \begin{pmatrix} \frac{1}{\beta} \\ \frac{1}{\alpha} \end{pmatrix}$$

$\lambda = -(\alpha+\beta)$

$$\begin{pmatrix} \alpha & \alpha \\ \beta & \beta \end{pmatrix}\begin{pmatrix} N_A^* \\ N_A \end{pmatrix} = \begin{pmatrix} 0 \\ 0 \end{pmatrix} \longrightarrow V_2 = \begin{pmatrix} 1 \\ -1 \end{pmatrix}$$

$$\begin{pmatrix} N_A^* \\ N_A \end{pmatrix} = C_1 \begin{pmatrix} \frac{1}{\beta} \\ \frac{1}{\alpha} \end{pmatrix} e^0 + C_2 \begin{pmatrix} 1 \\ -1 \end{pmatrix} e^{-(\beta+\alpha)t}$$

Applying the initial conditions at the moment lights are turned on, ie. All atoms are not excited

$$\begin{pmatrix} 0 \\ N_T - N_C \end{pmatrix} = \begin{pmatrix} \frac{C_1}{\beta} \\ \frac{C_1}{\alpha} \end{pmatrix} + \begin{pmatrix} C_2 \\ -C_2 \end{pmatrix} \longrightarrow \begin{matrix} 0 = \frac{C_1}{\beta} + C_2 \longrightarrow C_2 = -\frac{C_1}{\beta} \\ N_T - N_C = \frac{C_1}{\alpha} - C_2 = C_1\left(\frac{1}{\alpha} + \frac{1}{\beta}\right) = C_1 \frac{\beta+\alpha}{\alpha\beta} \end{matrix} \quad \begin{cases} C_1 = \dfrac{(N_T - N_C)\alpha\beta}{(\beta+\alpha)} \\ C_2 = \dfrac{-(N_T - N_C)\alpha}{(\beta+\alpha)} \end{cases}$$

$$N_A^* = -C_2 + C_2 e^{-(\beta+\alpha)t} = -C_2(1 - e^{-(\beta+\alpha)t})$$

$$N_A = -\frac{C_1}{\alpha} + \frac{C_1}{\beta} e^{-(\beta+\alpha)t} = C_1\left(\frac{1}{\alpha} + \frac{1}{\beta} e^{-(\beta+\alpha)t}\right)$$

Arriving to time dependent explicit formulas

$$N_A^* = \frac{(N_T - N_C)}{(\beta+\alpha)} \alpha(1 - e^{-(\beta+\alpha)t})$$

$$N_A = \frac{(N_T - N_C)\alpha\beta}{(\beta+\alpha)} \left(\frac{1}{\alpha} + \frac{1}{\beta} e^{-(\beta+\alpha)t}\right)$$

Under constant illumination equilibrium values of N_{A*} and N_A are stablished rapidly (in microsenconds or less). It is upon these “quasi-static” steady state values of N_{A*} and N_A that the slower oxidation process (seconds to hours) proceeds. Thus, the “quasi-static” values must not be interpreted as N_A and N_{A*} have constant values over the whole experiment. The equations explicitly show both depend on (N_T-N_C) which will slowly decrease as Nc (the oxidized $PbSO_x$) grows. All along the light emitted is always proportional to N_{A*} and a function of the radiative and non-radiative recombination paths.

In "steady state" →

$$N_A^* = \frac{(N_T - N_C)\alpha}{(\beta + \alpha)} \qquad N_A = \frac{(N_T - N_C)\beta}{(\beta + \alpha)}$$

$$\frac{N_A^*}{N_A} = \frac{\alpha}{\beta}$$

The number of oxidized atoms then can be calculated from:

$\frac{dN_C}{dt} = k^* N_A^* + \cancel{kN_A}$ (the kN_A term is much smaller and thus will be taken as zero)

$$\frac{dN_C}{dt} = \frac{k^*(N_T - N_C)\alpha}{(\beta + \alpha)}$$

$$\frac{dN_C}{dt} = BN_T - BN_c \quad \text{and} \quad B = \frac{k^*\alpha}{(\beta+\alpha)}$$

$$\int_0^{N_C} \frac{dN_C}{N_T - N_C} = \int_0^t Bdt'$$

$$-\ln(N_T - N_C)_0^{N_c} = Bt$$

$$-\ln(N_T - N_C) + \ln(N_T) = -Bt$$

$$\ln\left(\frac{N_T - N_C}{N_T}\right) = -Bt$$

$$\boxed{N_T - N_C = N_T e^{-Bt}}$$

Following our stated phenomenologial dependance of the non-radiative lifetime on the oxidation:

$$\tau_{NR} = \frac{1}{(N_T - N_C)^{\gamma}} = \frac{1}{(N_T e^{-Bt})^{\gamma}} \Rightarrow \tau_{NR} = \tau_{NR}^0 e^{\gamma Bt}$$

Allowing for the explict evoluton of τ_{NR} be used in the PL expression, then the PL yield can be compactly expressed as:

$$PL \sim \frac{N_A^*}{1+\tau_R/\tau_{NR}} = \frac{(N_T - N_C)\alpha}{(\beta+\alpha)(1+\frac{\tau_R}{\tau_{NR}^0 e^{\gamma Bt}})} = \frac{\alpha N_T e^{-Bt}}{(\beta+\alpha)(1+\frac{\tau_R}{\tau_{NR}^0}e^{-\gamma Bt})}$$

$$PL = M\frac{\alpha}{\beta+\alpha}\frac{e^{-Bt}}{\left(1+\frac{\tau_R}{\tau_{NR}^0}e^{-\gamma Bt}\right)} = A\frac{e^{-Bt}}{(1+Ce^{-\gamma Bt})}$$

M: microscope train constant

$$A = M\frac{\alpha}{\beta+\alpha};\ B = \frac{k^*\alpha}{(\beta+\alpha)};\ C = \frac{\tau_R}{\tau_{NR}^0}$$

Section b) numeric constants used in the model:

PbS pairs in 1 QD

$$\rho_{PbS} * V_{QD} = \text{Mass of QD}$$

$$(7.6\ {}^{g}/_{cm^3})\frac{4}{3}\pi(1.25\text{nm})^3 = 6.1 \times 10^{-20}\text{g}$$

$$6.1 \times 10^{-20}\text{g} = \text{M} * \text{n}$$

$$6.1 \times 10^{-20}\text{g} = \frac{239.3\ \text{g}}{\text{mol}} * \text{n}$$

$$2.59 \times 10^{-22}\text{mol} = \text{n}$$

$$2.59 \times 10^{-22} * \text{N}_\text{A} = \text{PbS pairs}$$

$$156 = \text{PbS pairs}$$

$$\sigma = \text{"area" of one PbS pair}$$

$$\sigma = \frac{1\ \text{QD in}\ 5 \times 5\ \text{nm}^2}{\text{PbS pairs in 1 QD}} \times 0.06$$

Experimental absorption

$$\sigma = \frac{25 \times 10^{-14}\text{cm}^2}{156\ \text{pairs}}$$

$$\sigma = 9.6 \times 10^{-17}\text{cm}^2$$

Thus, the α will be defined as:

$$\text{h}\upsilon = (2.33\ \text{eV})(1.6 \times 10^{-19}\frac{\text{J}}{\text{eV}})$$

$$\text{h}\upsilon = 3.72 \times 10^{-19}\text{J}$$

$$\alpha = \frac{\sigma \text{I}}{\text{h}\upsilon} = 258\frac{cm^2}{J} \times I$$

Section c) fit of other parameters:

On light intensity:

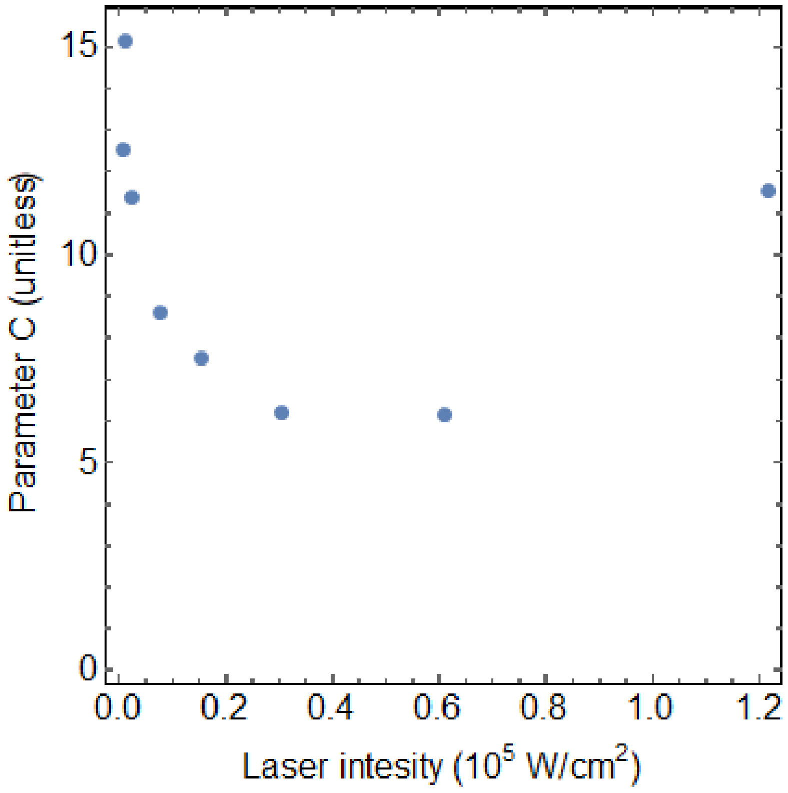

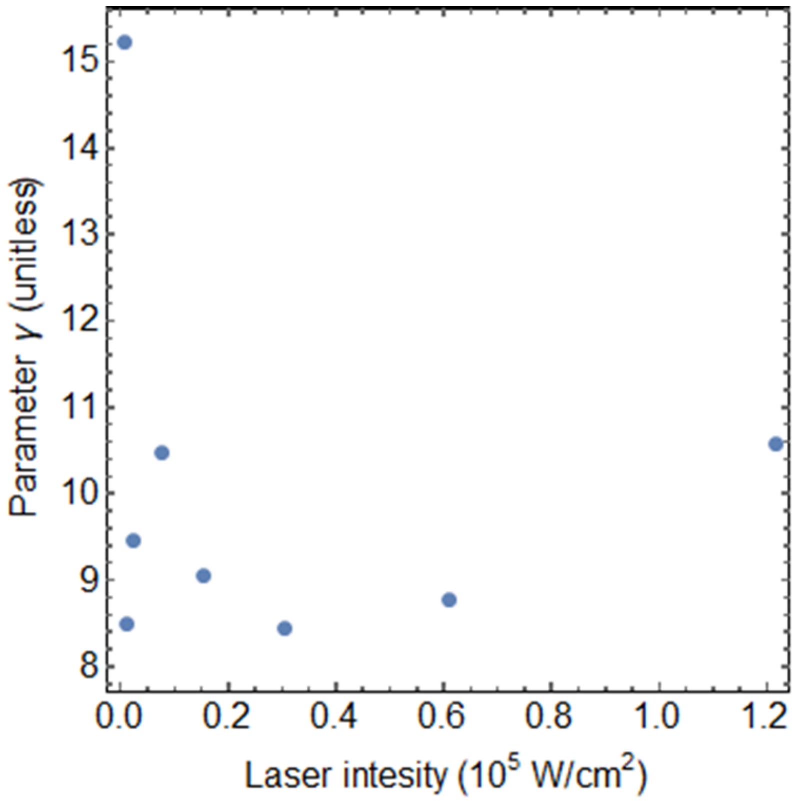

On gas pressure:

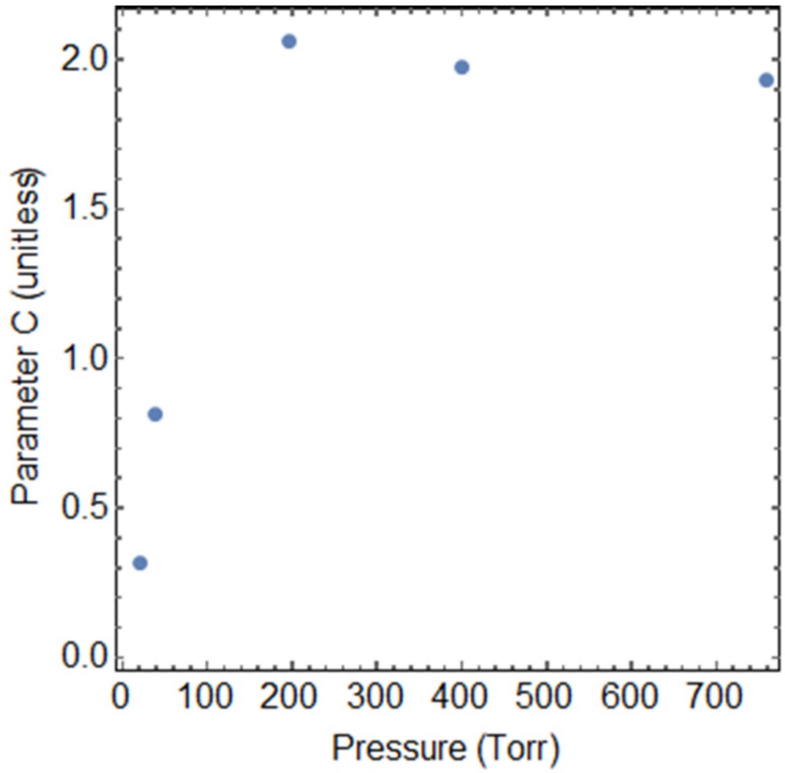

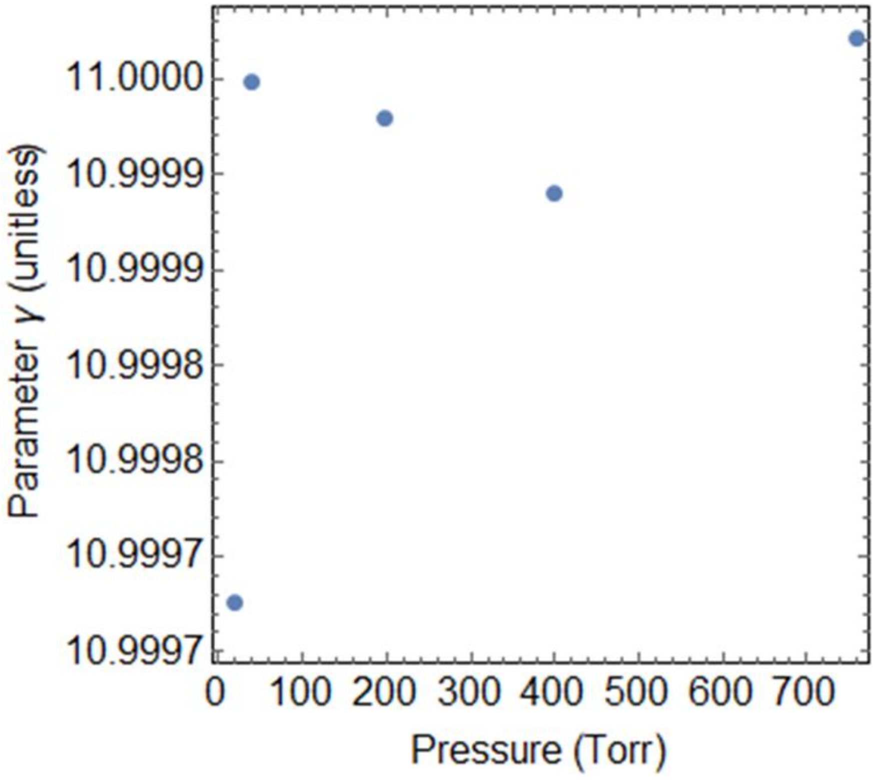

On temperature:

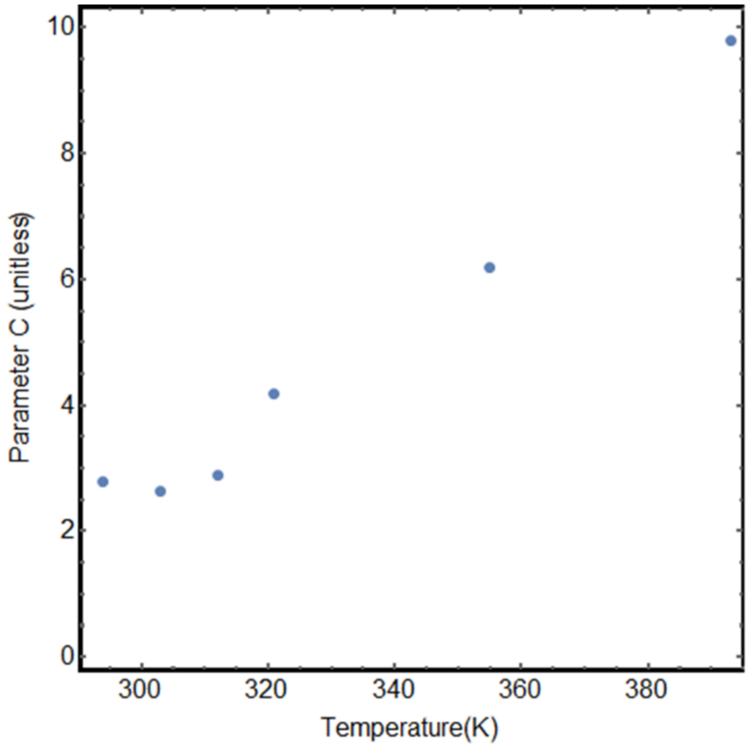

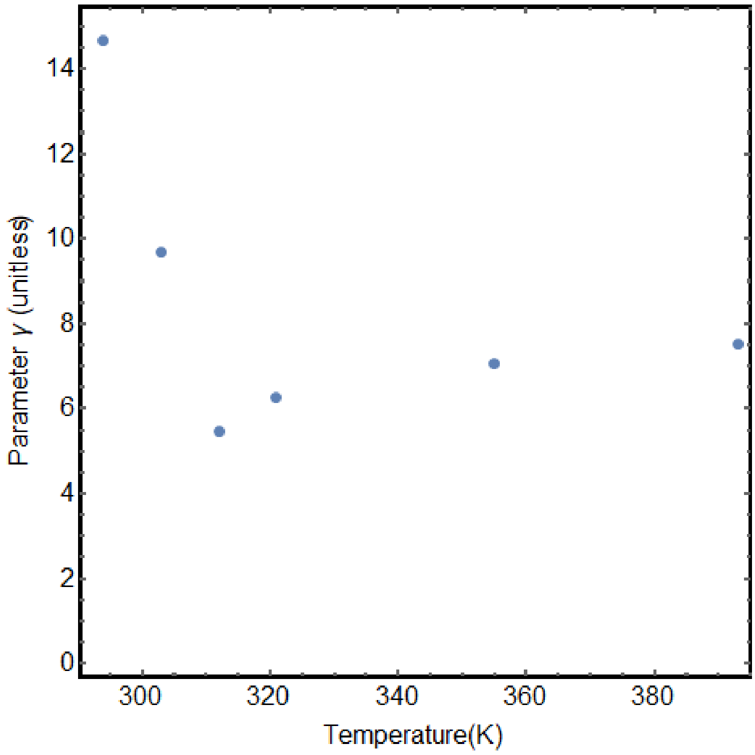

Supplement: Supplementary file 1 [file nanomaterials-08-00341-s001.pdf]
